# Iron and zinc micronutrients and soil inoculation of *Trichoderma harzianum* enhance wheat grain quality and yield

**DOI:** 10.3389/fpls.2022.960948

**Published:** 2022-09-07

**Authors:** Iftikhar Ali, Ajab Khan, Ahmad Ali, Zahid Ullah, Dong-Qin Dai, Naveed Khan, Asif Khan, Abdel Rahman Al-Tawaha, Hassan Sher

**Affiliations:** ^1^Center for Yunnan Plateau Biological Resources Protection and Utilization, Yunnan Engineering Research Center of Fruit Wine, College of Biological Resource and Food Engineering, Qujing Normal University, Qujing, China; ^2^Centre for Plant Science and Biodiversity, University of Swat, Charbagh, Pakistan; ^3^State Key Laboratory of Molecular Developmental Biology, Institute of Genetics and Developmental Biology, Chinese Academy of Sciences, Beijing, China; ^4^Laboratory of Phytochemistry, Department of Botany, University of São Paulo, São Paulo, Brazil; ^5^Department of Biological Sciences, Al Hussein Bin Talal University, Ma'an, Jordan

**Keywords:** wheat, *Trichoderma harzianum*, zinc, iron, HMW-GS

## Abstract

Malnutrition is mainly caused by iron and zinc micronutrient deficiencies affecting about half of the world's population across the globe. Biofortification of staple crops is the right approach to overcome malnutrition and enhance nutrient contents in the daily food of humans. This study aimed to evaluate the role of foliar application of iron and zinc in *Trichoderma harzianum* treated soil on various growth characteristics, quality, and yield of wheat varieties. Plants were examined in the absence/presence of *T. harzianum*, and iron and zinc micronutrients in both optimal and high-stress conditions. Although the symbiotic association of *T. harzianum* and common wheat is utilized as an effective approach for wheat improvement because of the dynamic growth promoting the ability of the fungus, this association was found tremendously effective in the presence of foliar feeding of micronutrients for the enhancement of various growth parameters and quality of wheat. The utilization of this approach positively increased various growth parameters including spike length, grain mass, biomass, harvest index, and photosynthetic pigments. The beneficial role of *T. harzianum* in combination with zinc and iron in stimulating plant growth and its positive impact on the intensities of high molecular weight glutenin subunits (HMW-GS) alleles make it an interesting approach for application in eco-friendly agricultural systems. Further, this study suggests a possible alternative way that does not merely enhances the wheat yield but also its quality through proper biofortification of iron and zinc to fulfill the daily needs of micronutrients in staple food.

## Introduction

Wheat (*Triticum aestivum* L.) belongs to the family Poaceae (grasses). It is an essential staple food and the most widely grown cereal crop in the world with a global annual production of more than 620 million tons (Mujeeb-Kazi et al., 2008). Approximately 36% of the world's population depends on wheat as a primary source of food due to the presence of 20% of the food calories, 55% carbohydrates (70–75% starch), 10–12% proteins, 14% water and lipids (2%) (Goel et al., 2021). Being staple food, wheat is mainly used as a source of food for humans while also utilized as a source of fodder for animals and as a substrate for mushroom cultivation (Shewry, 2009). The production of wheat is however severely affected by various biotic and abiotic factors (Waller et al., 2005). To encounter the need of a rapidly growing population, alternative approaches are required to enhance the wheat quality and yield (Cunha et al., 2022).

Micronutrients are required in lower amounts but are extremely vital for plant growth as compared to macronutrients (Tripathi et al., 2015). Deficiencies of micronutrients especially zinc (Zn) and iron (Fe) result in various diseases both in humans and plants (Pallavi and Sudha, 2017). In humans, micronutrient deficiencies are mainly due to their less intake in daily diet, while the growth and yield of plants are also affected when these micronutrients are less available in soil (Kihara et al., 2020). In cereal crops, Zn deficiency is a major problem because it reduces crop yields and human nutritional quality, particularly in those regions of the world which have a deficiency of Zn in soil (Cakmak and Kutman, 2018). Therefore, any increase in Zn and Fe concentrations in the cereal crops provides for healthy plants, enhancing crop yield and improving human health worldwide.

*Trichoderma harzianum* belongs to the genus Trichoderma and is characterized by rapid growth establishing a strong symbiotic association with many plant species (Visconti et al., 2020). *T. harzianum* can promote plant growth by enhancing the availability of nutrients to plants (Chun et al., 2018). It is present in almost every ecosystem and is involved in inhibiting plant pathogens in the soil to reduce plant diseases due to their mycoparasitic capacities (Hermosa et al., 2012; Guo Y. et al., 2019). Trichoderma has been used to protect different types of cereal crops from soil borne as well as air-borne diseases (Woo et al., 2014). Various species of Trichoderma improve plant growth by protecting them from bacteria and other pathogenic fungi (Błaszczyk et al., 2014).

Wheat has unique protein characteristics that serve as a vital resource of food and energy (Iftikhar and Ali, 2017). Among all cereals, wheat is the most extensively used crop accounting for about 20% of proteins and dietary calories required for human beings (Gupta et al., 2021). Glutenin, a type of storage protein in the wheat endosperm is considered to play a key role in controlling bread quality (Ali et al., 2013; Khalid et al., 2013; Ullah et al., 2016). Glutenin proteins contribute to 80–85% of the total wheat flour protein and are responsible for the viscoelastic properties of wheat flour and dough (Shewry et al., 1995). Further, the seeds of wheat contain proteins that determine the quality of bread making (Ali A. et al., 2015; Ali I. et al., 2015). Based on the mobility in SDS-PAGE (sodium dodecyl sulfate-polyacrylamide gel electrophoresis), glutenin proteins are divided into two subunits, high molecular weight glutenin subunits (HMW-GS) and low molecular weight glutenin subunits (LMW-GS) (Payne et al., 1979). Both LMW-GS and HMW-GS are a long chain of amino acids linked together by disulfide bonds forming macropolymer glutenin. Among them, HMW-GS plays an important role in controlling bread-making quality (Ali et al., 2013). Glutenin proteins can also be utilized as a biochemical marker for the identification and validation of wheat cultivars to check the purity of seed (Ullah et al., 2016), and to determine the quality of wheat products and human nutrition (Xu et al., 2012).

Wheat growth and development are affected by various biotic and abiotic factors. Further, high increase in population growth, industrialization, stagnant wheat yield per unit area, lack of essential micro-macronutrients, the use of uncertified seeds and pathogens, and pest attacks significantly increase the wheat production requirement globally (Ali et al., 2014; Seleiman et al., 2021; Erenstein et al., 2022; Reynolds and Braun, 2022).

Nutrient foliar application is considered an important approach to enhancing crop yields and grain micronutrient content. Several studies have demonstrated that zinc and iron foliar application resulted in enhanced growth and yield characteristics of wheat crops (Cakmak et al., 2010; Li et al., 2016). However, little is known about the combined effect of Zn and Fe on the quality traits of wheat kernels. The current study was therefore conducted to investigate the combined effect of zinc, iron, and *T. harzianum* on wheat growth, yield attributes, and grain quality with a focus on the intensity of HMW-GS alleles.

## Materials and methods

### Plant materials, growth conditions, and treatments

The research was conducted at the Center for Plant Sciences and Biodiversity, University of Swat, Khyber Pakhtunkhwa, Pakistan. The experimental site is located at 34°3'N and 71°50'E, 940 m asl with an annual rainfall of 737.3 to 1,200 mm and an average temperature from 11.3 to 25.7°C. Detailed characterization of the experimental soil revealed its texture as silt loam with 3.2% sand, 26.7% clay, and 70.1% silt; having 1.2% organic matter; pH 6.4; EC 0.17 m.mohs cm^−1^; noncalcareous and non-saline in nature. The recommended fertilizer dose of 20–60–60 kg ha^−1^ (NPK) was applied before sowing. Healthy and clean seeds of Pirsabak 2005 were sown in 3 m long rows with 30 cm inter-row spacing which formed an experimental unit/plot. Compost was administered to all plots at the rate of 20 tons ha^−1^. The conidia suspension of *T. harzianum* strain G8 was prepared according to the method of Zhang et al. (2014). G8 strain obtained from the Institute of Genetics and Developmental Biology, Chinese Academy of Sciences, Beijing, China was cultured at 25°C for 1 week in Petri dishes containing potato dextrose agar (PDA) solid medium. The conidia suspension of *T. harzianum* was then made by adding 5 ml of sterilized distilled water to the surface of the agar and the collected suspension was transferred into 500 ml beakers. The suspension was then adjusted to 100 ml, the conidia were separated and dispersed by a hand mixer and the conidia density was determined by a hemocytometer and adjusted to 1.5 × 10^7^ conidia per ml. Then 1 ml of the conidia suspension of *T. harzianum* was inoculated into 60 ml of the sterilized liquid culture medium of potato dextrose broth (PDB) in a 150 ml flask. Then the flask was kept at 30°C for 9 days in a shaking incubator running at 120 rpm. The fungi were purified, and the spore suspension produced was inoculated in fresh PDB containing a drop of Tween-80. The spores were collected in sterile water and were filtered through a Whatman Paper and then through 0.22 mm Millipore membranes. The concentration was then adjusted to 1.5 × 10^7^ spores per ml and was used for treatment. At a pre-anthesis stage, following Sesan et al. (2020) with minor modification as per the requirement of the experiment, micronutrients including zinc as ZnSO_4_ (zinc sulfate) and iron as FeSO_4_ (iron sulfate) were applied as foliar spray alone and in combination with *T. harzianum*, each in two concentrations at the rate of 9 and 18 mm, respectively. A total of 12 treatments were applied which comprised of *control*; *T.harzianum*; *Zn-9mM*; *Fe-9mM*; *Zn-18mM*; *Fe-18mM*; *Fe-9mM/T.harzianum*; *Fe-18mM/ T.harzianum*; *Zn-9mM/T.harzianum*; *Zn-18mM/T.harzianum*; *Fe-9mM)/T.harzianum/Zn-9mM*; and, *Fe-18mM/T.harzianum/ Zn-18mM*. The layout of the experiment followed a randomized complete block design having 3 replications (Li et al., 2016).

### Chlorophyll measurement

After 10 days of applying these treatments, fresh leaves were collected from every plot to measure chlorophyll quantity following the method of Hiscox and Israelstam (1979). Fresh leaves of 0.5 g were collected, wrapped in aluminum foil, and kept in the freezer for 72 h. Weighed leaf tissue in fractions was taken in a tube containing 6 ml dimethyl sulfoxide (DMSO) preheated to 65°C in a water bath for 30–40 min. Then 4 ml DMSO was added to all samples to make the final volume 10 ml. Finally, the solution extracted from leaves was observed in 645 and 663 nm with a spectrophotometer (BMS-UV 1602). Chlorophyll *a, b*, and total chlorophyll contents were calculated by using the equations of Arnon's (1949).

### Determination of phenological traits

Phenological traits including days to heading (DH), days to physiological maturity (PM), spike length (SPL), seeds per spike (SPS), and grain mass (GM) were recorded as discussed in Afzal et al. (2018). Biomass and harvest index of the experimental plant material was also determined as described in Dreisigacker et al. (2021).

### Analysis of high molecular weight glutenin sub-units

Identification of HMW-GS of the studied wheat was accomplished by crushing a single kernel from each treatment, followed by protein extraction and SDS-PAGE analysis according to Ali et al. (2013). The SDS-PAGE gel was stained in a solution containing 44% methanol, 6% acetic acid, and 0.22% Coomassie brilliant blue R350, while, de-stained in a solution of 20% methanol and 5% acetic acid. The allelic diversity at different locus *Glu-A1, Glu-B1*, and *Glu-D1* was determined using 12 different quality standards obtained from CIMMYT, Mexico (Ullah et al., 2016). The intensity of HMW-GS under different treatments was also measured by a computer software image J. For this purpose, the de-stained gel was scanned with a high-resolution scanner, converted to a grayscale image, and processed as previously described in Ali et al. (2020) and Bonilla et al. (2020).

### Statistical analysis

The data were recorded in triplicates for each treatment and analyzed by one-way analysis of variance. Multiple Duncan range test (DMRT) using SPSS version 21.0 depicted the differences among treatments at *p* < 0.05 level.


$$\begin{array}{l} \%\;\left(increase\;or\;decrease\right)=\frac{Contorl-Treatment}{Control}\times 100 \end{array}$$

## Results

The foliar application of Zn and Fe, either alone or in combination with *T. harzianum*, enhanced all the studied attributes in the experimental wheat. Analysis of variance depicted differences among treatments with respect to the studied traits including days to heading (DH), physiological maturity (PM), plant height (PH), spike length (SPL), grain mass (GM), seeds per spike (SPS), chlorophyll-a (Chla), total chlorophyll (TChl) and biomass (Table 1; Supplementary Table S1).

**Table 1:** Mean squares of the different traits in the studied wheat genotype.

**SOV**	**DF**	**DH**	**PM**	**PH**	**SPL**	**Biomass**	**GM**	**HI**	**SPS**	**Chl a**	**Chl b**	**TChl**
Treatment	11	4.87***	605.1***	260.6***	4.646***	170939***	12303.2***	0.0003^NS^	49.60*	0.010***	0.027^NS^	0.053***
Error	22	1.202	2.629	13.80	0.167	24976	3191.0	0.0004	16.59	0.003	0.009	0.015

where, PM, physiological maturity; SPS, seeds per spike; PH, plant height; SPL, spike length; DH, days to heading; HI, harvest index; GM, grain mass; Chl a, chlorophyll a; Chl b, chlorophyll b; TChl, total chlorophyll. *, Significant, ***, Highly significant and NS, Non-significant.

**Figure 1:**
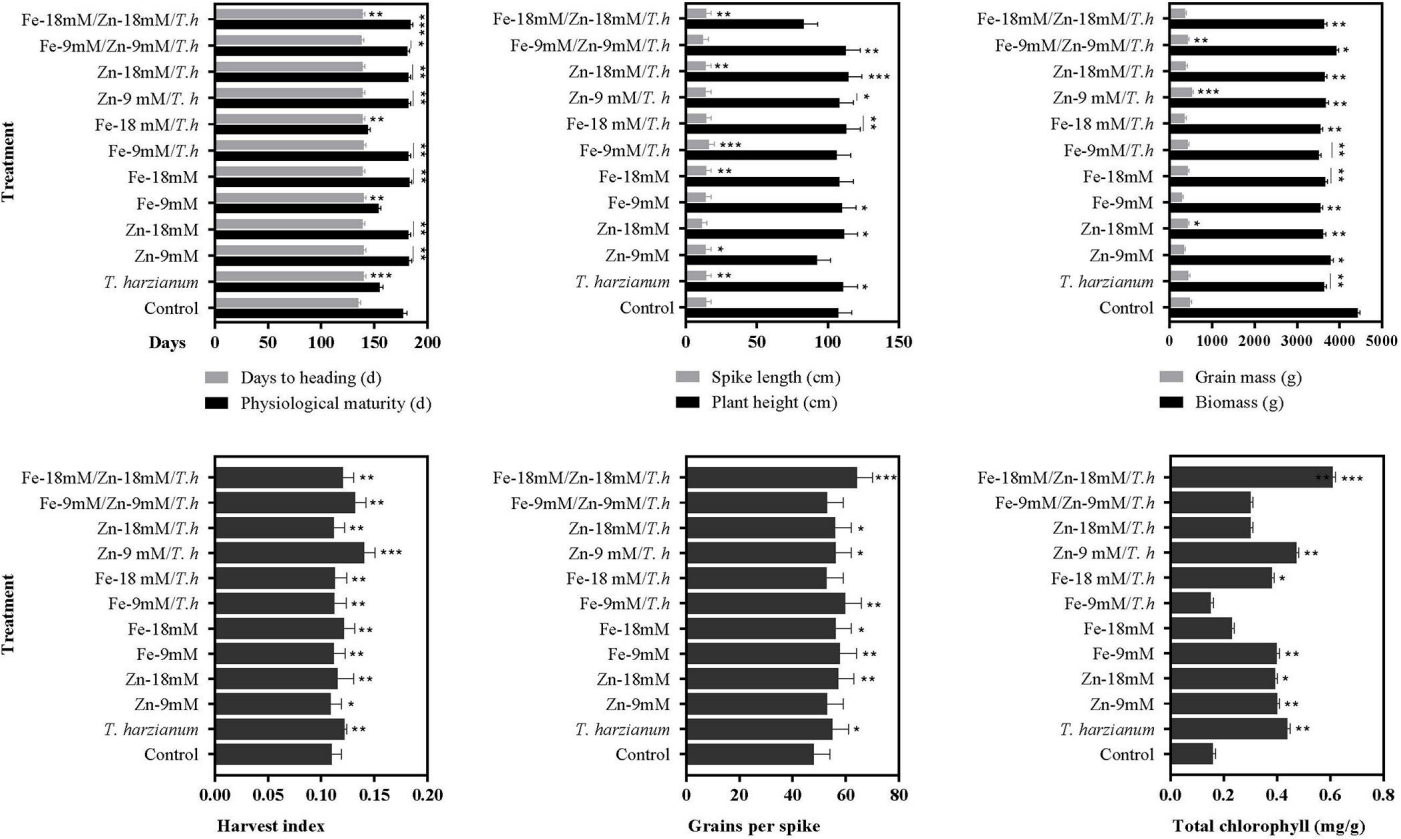
Effects of Fe, Zn, and *Trichoderma harzianum* on the studied traits. Where: *, ** and *** are depicting statistical significances at 0.05, 0.01, and 0.001 probability level, respectively. While the bars without * are showing no statistically significant differences (*NS*).

The studied wheat also exhibited differential behavior in response to foliar application of micronutrients and *T. harzianum* (Figure 1; Table 2; Supplementary Table S2). It was observed that treatment either alone or in combination significantly stimulated days to heading, and a maximum enhanced effect of 3.6% was found in wheat plants treated with *T. harzianum*, while the least effect was noted in plants treated with *Fe-9mM/T.harzianum/Zn-9mM* when compared with control treatment. Regarding days to physiological maturity, the maximum effect (3.2%) was recorded for treatment *Fe-18mM/T.harzianum/Zn-18mM*. However, PM was negatively affected by treatment *Fe-18mM/T.harzianum* with an observed decrease of 18.9% when compared to plants grown in control conditions. Similarly, in comparison with control conditions, all the treatments resulted in enhanced plant height, however, the most effective amongst all the treatments was *Zn-18mM/T.harzianum* which led to the maximum recorded enhanced PH of 6.3%. The effect of other treatments including *Fe-18mM/T.harzianum/Zn-18mM* and *Zn-9mM* was negative which decreased PH by 22.7 and 13.8%, respectively. Concerning spike length, the treatment *Fe-9mM/T.harzianum* was much more promising as it enhanced SPL by 12.2%, while other treatments negatively affected the same studied trait which included *Zn-18mM* (21.8%), *Fe-9mM/T.harzianum/Zn-9mM* (15.1%) and *Zn-9mM/T.harzianum* 3.9%). The maximum harvest index was exhibited in the plants treated with *Zn-9 mM/T.harzianum*. While, as compared to control the least harvest index was exhibited in the plants treated with *Zn-9mM*. Similarly, the current research showed that maximum seeds per spike were exhibited by plants treated with *Fe-9mM/T.harzianum*.

**Table 2:** Analysis of variance of different traits in studied wheat genotype.

**Treat**	**DH**	**PM**	**PH**	**SPL**	**Biomass**	**GM**	**HI**	**SPS**	**Chla**	**Chlb**	**Tch**
	**(d)**	**(d)**	**(cm)**	**(cm)**	**(g)**	**(g)**			**(mg/g)**	**(mg/g)**	**(mg/g)l**
Control	135.3^c^	178.0^c^	107.2^bc^	14.37^b^	4416.7^a^	478.7^ab^	0.108^ab^	47.8^d^	0.100^c^	0.033^c^	0.160^d^
*T.harzianum*	140.3^a^	154.7^d^	110.8^abc^	14.33^b^	3630.7^b^	440.7^abc^	0.122^ab^	55.0^bc^	0.253^ab^	0.187^abc^	0.440^abc^
Zn-9mM	139.7^ab^	182.0^ab^	92.4^d^	13.80^b^	3788.7^ab^	339.0^de^	0.106^b^	52.8^bcd^	0.230^ab^	0.173^abc^	0.403^abc^
Zn-18mM	138.7^ab^	182.3^ab^	111.5^abc^	11.23^d^	3614.7^b^	417.3^bcd^	0.116^ab^	57.3^abc^	0.260^ab^	0.136^bc^	0.396^bc^
Fe-9mM	139.7^ab^	154.3^d^	109.7^abc^	14.10^b^	3538.0^b^	282.3^e^	0.116^b^	57.6^abc^	0.256^ab^	0.143^bc^	0.400^abc^
Fe-18mM	139.3^ab^	183.3^ab^	108.0^bc^	14.33^b^	3654.7^b^	422.7^abcd^	0.124^ab^	56.2^bc^	0.193^bc^	0.046^c^	0.233^cd^
Fe-9mM)/*T.harzianum*	139.7^ab^	182.3^ab^	106.1^c^	16.37^a^	3509.3^b^	422.3^abcd^	0.118^ab^	59.6^ab^	0.123^c^	0.063^c^	0.153^d^
Fe-18mM/*T.harzianum*	139.0^ab^	144.3^e^	112.9^ab^	14.23^b^	3544.7^b^	353.0^cde^	0.119^ab^	52.6^cd^	0.253^ab^	0.130^bc^	0.383^bc^
Zn-9mM/*T.harzianum*	139.3^ab^	181.7^ab^	108.3^abc^	13.80^b^	3674.0^b^	516.7^a^	0.142^a^	56.3^bc^	0.247^ab^	0.230^ab^	0.476^ab^
Zn-18mM/*T.harzianum*	138.7^ab^	182.3^ab^	114.4^a^	14.10^b^	3642.7^b^	378.7^cd^	0.115^ab^	55.8^bc^	0.230^ab^	0.073^bc^	0.303^bcd^
Fe-9mM/*T.harzianum*/Zn-9mM	138.0^b^	180.7^bc^	112.6^ab^	12.20^c^	3921.3^ab^	424.3^abcd^	0.135^ab^	52.8^bcd^	0.190^bc^	0.110^bc^	0.303^bcd^
Fe-18mM/*T.harzianum*/Zn-18mM	139.0^b^	184.0^a^	82.9^e^	14.30^b^	3639.3^b^	359.7^cde^	0.121^ab^	64.2^a^	0.297^a^	0.317^a^	0.610^a^

For traits code, see Table 1. Different letters are showing statistical differences among treatments at *p* < 0.05.

### Chlorophyll contents

**Figure 2:**
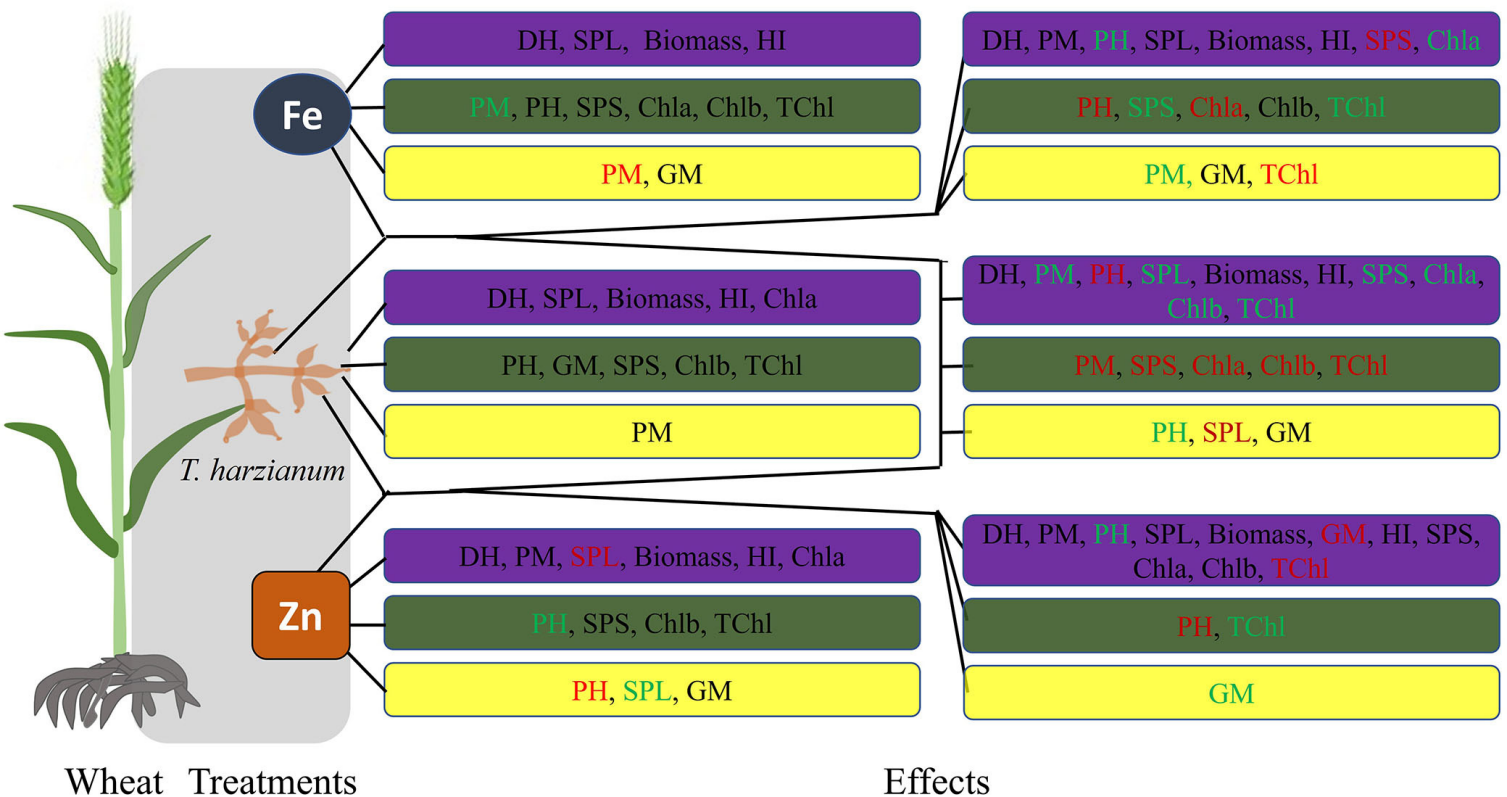
Overview of wheat responses for the studied traits to different treatments. Blue boxes represent enhanced effect; green boxes represent no significant differences; while, yellow boxes represent negative/decreased effect of the respective treatment, in comparison to control. Further, the black font color of the studied trait represents a response to both low and high concentration; the red color represents a response to low concentration; while, the green color represents a response to the high concentration of the respective treatment.

As compared to the control, the chlorophyll contents increased with the application of Zn, Fe, and *T. harzianum* (Figures 1, 2; Table 2). Likewise, chlorophyll contents were significantly increased in wheat plants treated with the *Fe-18mM/T.harzianum* and *Zn-18mM/T.harzianum*. It is pertinent to mention that chlorophyll content remained almost unaffected in response to both foliar applications of Zn and Fe. On the contrary, the treatment *Fe-9mM/T.harzianum* resulted in a slight decrease in total chlorophyll content. However, its combination with *T. harzianum* significantly enhanced chlorophyll content, but only at a high concentration (*18mM*).

### HMW-GS determination

**Figure 3:**
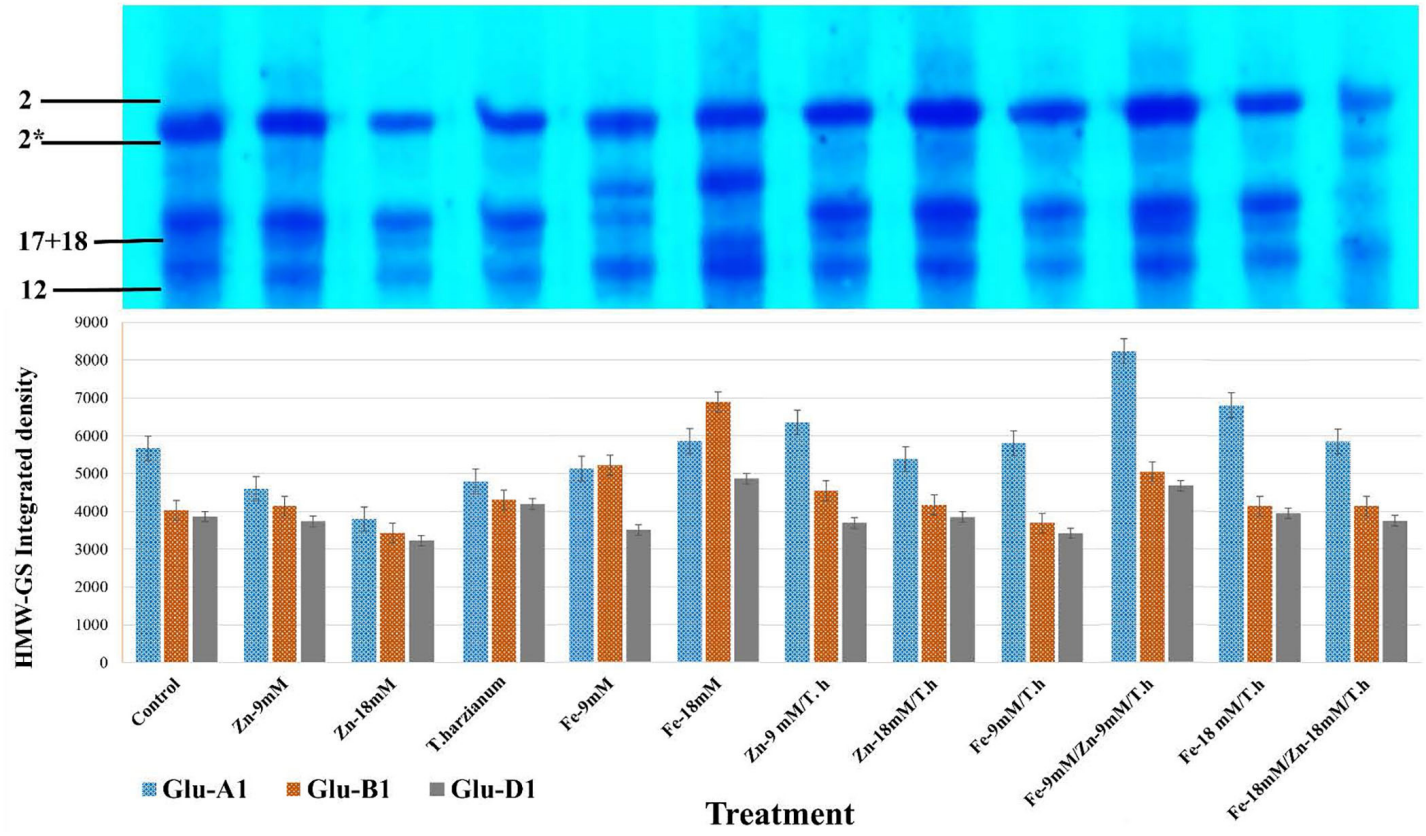
SDS-PAGE profile of HMW-GS alleles of the studied wheat cultivar and its area integrated densities under different treatments. For accuracy and validity of data, the average area integrated densities were determined for comparison. HMW-GS 2* asterisk.

In the currently studied wheat genotype (Pirsabak 2005), the observed compositions of HMW-GS were 2^*^ (encoded by *Glu-A1 b*), 17+18 (encoded by *Glu-B1i*), and 2+12 (encoded by *GLUD1a*) (Figure 3; Supplementary Figure S1; Supplementary Table S2). This is denoted that in wheat, HMW-GS are coded by co-dominant genes *GluA1, Glu-B1*, and *Glu-D1* which are present at *Glu-1* loci on the long arms of the homologous group 1 chromosome. For correct scoring of HMW-GS 12 different standards were selected and were used in SDS-PAGE along with our samples. Variable densities of HMW-GS alleles were found in response to different foliar treatments (Figure 3). For the accuracy and validity of the observed data, the average area integrated densities were determined from three different replicates. The observed densities of *Glu-A1* were enhanced by all the treatments except *Zn-9mM, Fe-9mM*, and *Zn-18mM/T.harzianum*. In comparison to control treatment, maximum increased densities were depicted by *Fe-9mM/T.harzianum/Zn-9mM* (31.1%), *Fe-18mM/T.harzianum* (16.7%), *Zn-9mM/T.harzianum* (10.8%) and *Fe-18mM* (3.3%). Regarding *Glu-B1* alleles, increased densities were observed for the treatments *Fe-18mM* (41.6%), *Fe-9mM* (22.8%), *Fe-9mM/T.harzianum/Zn-9mM* (20.1%), *Zn-9mM/T.harzianum* (11.4%) and *T.harzianum* (6.3%). While the treatments *Zn-18mM* and *Fe-9mM/T.harzianum* negatively affected its relative densities. Similarly, with respect to *Glu-D1*, the treatments *Fe-18mM* (20.6%), *Fe-9mM/T.harzianum/Zn-9mM* (17.3%), *T.harzianum* (7.9%) and *Fe-18mM/T.harzianum* (2.2%) led to increased densities in comparison to control.

## Discussion

Biofortification is a suitable approach to improving crop yield and the quality of human diet (White and Broadley, 2009). Particularly in areas having malnutrition, this current research identified new approaches that will result in the improvement of the nutritional quality of crops without reducing yields (Aziz et al., 2019). Minerals nutrition plays an important role in the plant's physio-molecular traits including photosynthesis, gene expression, carbohydrate accumulation, enzymatic activities, signal transduction pathways, respiration, and recovery from stress conditions (Li et al., 2016). However, the quantity of nutrients (zinc and iron) requirement considerably depends on its quantity as well as its available form in the soil, cultivar characteristics, and yield (Rashid and Ryan, 2004). The unavailability of micronutrients in the soil might be due to its complex form (chelation), access to CaCO_3_, organic matter, and pH of the soil (Salim and Raza, 2020). Foliar application of micronutrients is one of the best and most suitable approaches which can provide a practical way for the improvement of cereal crop yield with balanced nutrients (Aziz et al., 2019). Further, another approach to increase mineral availability to plants includes the use of plant growth promoting microbes (PGPM), which significantly improve the yield attributes of the wheat crop, like plant height, dry and fresh weight, net photosynthesis, grain yield, spike length and other physiochemical parameters of wheat plants (El-Katatny and Idres, 2014).

Foliar application of iron and zinc alone and in combination with *T. harzianum* significantly affected various growth traits of the studied wheat cultivar including increased leaf chlorophyll contents, spike length, plant height, biomass, and grain mass. This may be attributed to the potential promising role of Zn and Fe in the grain filling and reproductive stages of wheat as previously discussed by Zain et al. (2015) and Yaseen et al. (2018). Our findings are in general agreement with those reported by El-Katatny and Idres (2014), Colla et al. (2015), and Sani et al. (2020), which further support the potential effects of these micronutrients in combination with *T. harzianum* to enhance plant overall performances. Our results are also in general agreement with those reported by Jalal et al. (2020) who found that foliar application of Zn in combination with Fe significantly improved grain yield, grain nutrients, and biomass. Higher yield in the studied wheat under foliar application of micronutrients may be due to better availability of these micronutrients, as these nutrients may be less available when applied to soil due to their immobility in alkaline soils. Physio-biochemical roles of Fe and Zn in plant growth have been previously reported by Hanjagi and Singh (2017). Significant roles of Zn and Fe in the improvement of wheat physiology have also been reported by Babaeian et al. (2011) and Impa et al. (2019). Another reason for higher yield could be due to the presence of environmentally friendly microbe i.e., *Trichoderma* which can enhance crop quality and quantity in an eco-friendly manner (Ogut and Er, 2006). In contrast, in the access quantity, these minerals could also form chelates not easily available to plants though Trichoderma helps in the conversion of them into the simplest form due to which the soil becomes more fertile resulting in better plant growth and development (Visconti et al., 2020).

Micronutrients, specifically Zn and Fe have a significant role in plant physiology and biochemical metabolism (Moshfeghi et al., 2019). For instance, Hermosa et al. (2012) reported that the supply of zinc in proper amounts increased plant physiological activities. Previously it was also reported by Ma et al. (2017) that under adverse conditions, such as salt and drought stress, application of Zn and Fe significantly increased photosynthetic pigments, photosynthesis rate, and overall growth of plants. Soil or foliar application of Fe and Zn separately or in combination increased plant height, tiller number, spike length, seeds per spike, 1,000 grain weight, biomass, and harvesting index in wheat plants (Piri, 2012; Ramzan et al., 2020).

However, the availability of these micronutrients in alkaline and calcareous soils become insufficient and often leads to a reduction in plant growth (Gentili et al., 2018). As Fe and Zn have limited mobility inside the plant body, adding them directly over parts of the plants is a good strategy. Foliar applications can serve this purpose very efficiently. An increase in the uptake of micronutrients by foliar application in the current study indicated the effectiveness of the foliar application. Yaseen et al. (2018) suggested that this approach is particularly true for saline and alkaline soils. Moreover, an increase in the uptake of micronutrients can enhance the uptake of macronutrients in plants (Abbas et al., 2009).

When compared to control, the growth traits like spike length and plant and grain biomass were non-significantly affected. A similar finding was reported by Sahin and Işler (2021) where Zn foliar spray did not significantly increase the soybean quantity and quality. In contrast, Hosseinzadeh and Ahmadpour (2018) reported that Zn foliar spray somehow resulted in enhanced growth and development, especially at the flowering stage. Our results about spike length are in general agreement with those described by Abbas et al. (2009) who found that application of Zn significantly increased spike length. Similarly, Zain et al. (2015) also found that spike length was highly increased when micronutrients (ZnSO4 and FeSO4) were applied as a foliar application. Likewise, Afshar et al. (2020) also reported that foliar application of Zn meaningfully increased spike length than control. However, the reason could be due to the long-term exposure of plants to any sort of stress that adversely affect the growth and development of plants. It is one of the key factors causes that affect soil properties, making it more acidic, impacting cation exchanges and soil structure, and influencing microorganism's beneficial capabilities and useful oxidation-reduction reactions.

Photosynthesis in plants is regulated by several factors like nutrient availability, pH of the soil, light, water, heat, and carbon dioxide (Guo J. et al., 2019). These factors interfere directly and indirectly by altering the enzymatic and chemical processes in the photosynthesis of plants. In the current study, the chlorophyll contents were significantly improved with the application of Fe, Zn, and *T. harzianum* combined treatment. It has been hypothesized that Zn and Fe improved the synthesis of chlorophyll because they act as a structural component as well as a catalytic component of enzymes and proteins, and are also essential for the normal synthesis of photosynthetic pigment (Wani et al., 2022). Trichoderma sp. was previously reported to have significant impacts on drought stress, particularly *T. harizianum* have been reported to have beneficial applications for rice drought tolerance (Shukla et al., 2012; Khadka and Uphoff, 2019; Hewedy et al., 2020).

Our results are in agreement with the previous findings of Colla et al. (2015), who found that arbuscular mycorrhizal fungi have the capabilities of root colonization that ultimately increase the chlorophyll biosynthesis, functions, and efficacy, which could be associated with higher net CO_2_ assimilation rate in the plant's growth and development. Another possible reason could be the availability of maximum chlorophyll contents and micronutrients which are the basic structural and functional components, which they colonize during the photosynthetic organelle's biosynthesis (Moshfeghi et al., 2019; Niyigaba et al., 2019; Szerement et al., 2021).

The observed composition of HMW-GS for the studied wheat cultivar was 2^*^, 17 + 18, and 2 + 12. Further, variable integrated densities of HMW-GS alleles were found in response to different foliar treatments. For instance, in *Glu-A1*, maximum increased densities were depicted by *Fe-18mM/T.harzianum, Fe-9mM/T.harzianum/Zn-9mM, Zn-9mM/T.harzianum* and *Fe-18mM*, in comparison to control. Similarly, in *Glu-D1*, treatments including *Fe-18mM, T.harzianum, Fe-9mM/T.harzianum/Zn-9mM*, and *Fe-18mM/T.harzianum* led to increased densities in comparison to control. These results predict a potentially significant role of the provided treatments in cultivating wheat for different quality traits. For example, it has been reported that glutenin strength increases with the HMW-GS allelic combination of 5 + 10, 17 + 18, and 2^*^ (Song et al., 2020). Similarly, Maryami et al. (2020) observed that *Glu-A1* alleles including 2^*^ were associated with higher gluten quality than the null allele and that *Glu-B1* encoded subunit 17 + 18 was highly correlated with bread texture, volume, and cell traits. The beneficial role of *T. harzianum* in combination with zinc and iron in stimulating plant growth, efficient use of micronutrients, and its positive impact on the intensities of HMW-GS alleles make it interesting as an alternate approach for application in eco-friendly agricultural systems (Vukelic et al., 2021; Ali et al., 2022).

## Conclusion

Application of *T. harzianum* in combination with foliar applied zinc and iron significantly enhanced plant height, spike length, grain yield, number of seeds per spike, and harvest index specifically in treatments *Zn-9mM, Zn-18mM, Fe-9mM/T.harzianum/Zn-9mM*, and *Fe-18mM*. Photosynthetic pigments such as chlorophyll a, chlorophyll b, and total chlorophyll were also improved with the application of micronutrients in combination with *T. harzianum*. The current study could be used successfully for bread wheat development programs and may serve to meet the need for bread wheat with enhanced yield attributes and appropriate gluten strength.

## Data availability statement

The original contributions presented in the study are included in the article/Supplementary material, further inquiries can be directed to the corresponding authors.

## Author contributions

AA and ZU designed the research. IA, AjK, and NK conducted the research. IA, AA, AjK, and AsK analyzed the data. HS, D-QD, and AA-T checked and revised the final content of the manuscript. All authors contributed to the article and approved the submitted version.

## Funding

This research was supported by the National Natural Science Foundation of China (No. NSFC 31760013) and High-Level Talent Recruitment Plan of Yunnan Provinces Young Talents Program.

## Conflict of interest

The authors declare that the research was conducted in the absence of any commercial or financial relationships that could be construed as a potential conflict of interest.

## Publisher's note

All claims expressed in this article are solely those of the authors and do not necessarily represent those of their affiliated organizations, or those of the publisher, the editors and the reviewers. Any product that may be evaluated in this article, or claim that may be made by its manufacturer, is not guaranteed or endorsed by the publisher.
