# Supplementary material for: Iron and zinc micronutrients and soil inoculation of Trichoderma harzianum enhance wheat grain quality and yield
Source: Front Plant Sci. 2022 Sep 7;13:960948. doi: 10.3389/fpls.2022.960948 (PMC9490233; doi:10.3389/fpls.2022.960948)
Supplement: Supplementary file 1 [file Data_Sheet_1.docx]

**Table S1.** Summary statistics of the studied traits

|  | **DH**  **(d)** | **PM (cm)** | **PH (cm)** | **SPL (cm)** | **Biomass**  **(g)** | **GM**  **(g)** | **HI** | **SPS** | **Chl a (mg/g)** | **Chl b (mg/g)** | **TChl (mg/g)** |
| --- | --- | --- | --- | --- | --- | --- | --- | --- | --- | --- | --- |
| **Mean** | 138.9 | 174.2 | 106.4 | 13.93 | 3714.6 | 402.9 | 0.120 | 55.67 | 0.219 | 0.137 | 0.355 |
| **Variance** | 2.444 | 191.9 | 90.80 | 1.570 | 16484 | 6003.2 | 0.0003 | 27.73 | 0.005 | 0.014 | 0.083 |
| **S.E Mean** | 0.261 | 2.310 | 1.590 | 0.210 | 67.70 | 12.91 | 0.003 | 0.880 | 0.012 | 0.020 | 0.0281 |
| **C.V** | 1.123 | 7.950 | 8.950 | 9.010 | 10.93 | 19.23 | 16.64 | 9.460 | 32.92 | 85.53 | 47.38 |
| **Minimum** | 135.0 | 144.0 | 81.90 | 10.70 | 3008.0 | 249.0 | 0.073 | 44.70 | 0.050 | 0.010 | 0.110 |
| **Maximum** | 142.0 | 185.0 | 116.8 | 16.90 | 4550.0 | 606.0 | 0.156 | 68.80 | 0.320 | 0.440 | 0.740 |

For traits code, see Table 1

**Table S2.** HMW-GS subunit composition of studied genotype**.**

| **S.No.** | **Genotype** | **Subunit composition** |
| --- | --- | --- |
| 1 | Pirsabak-05 | 2*; 17+18; 2+12 |


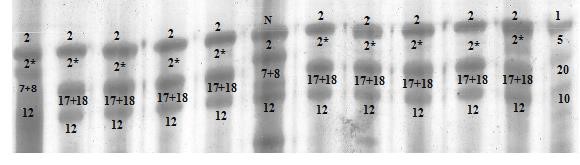


**Figure S1: SDS-PAGE profile of HMW-GS alleles of wheat genotype.** Lane from Left: 1: Darius, 2, 3, 4, 5: Pirsabak-05, 6: CS, 7, 8, 9, 10, 11: Pirsabak-05, 12: Halberd.


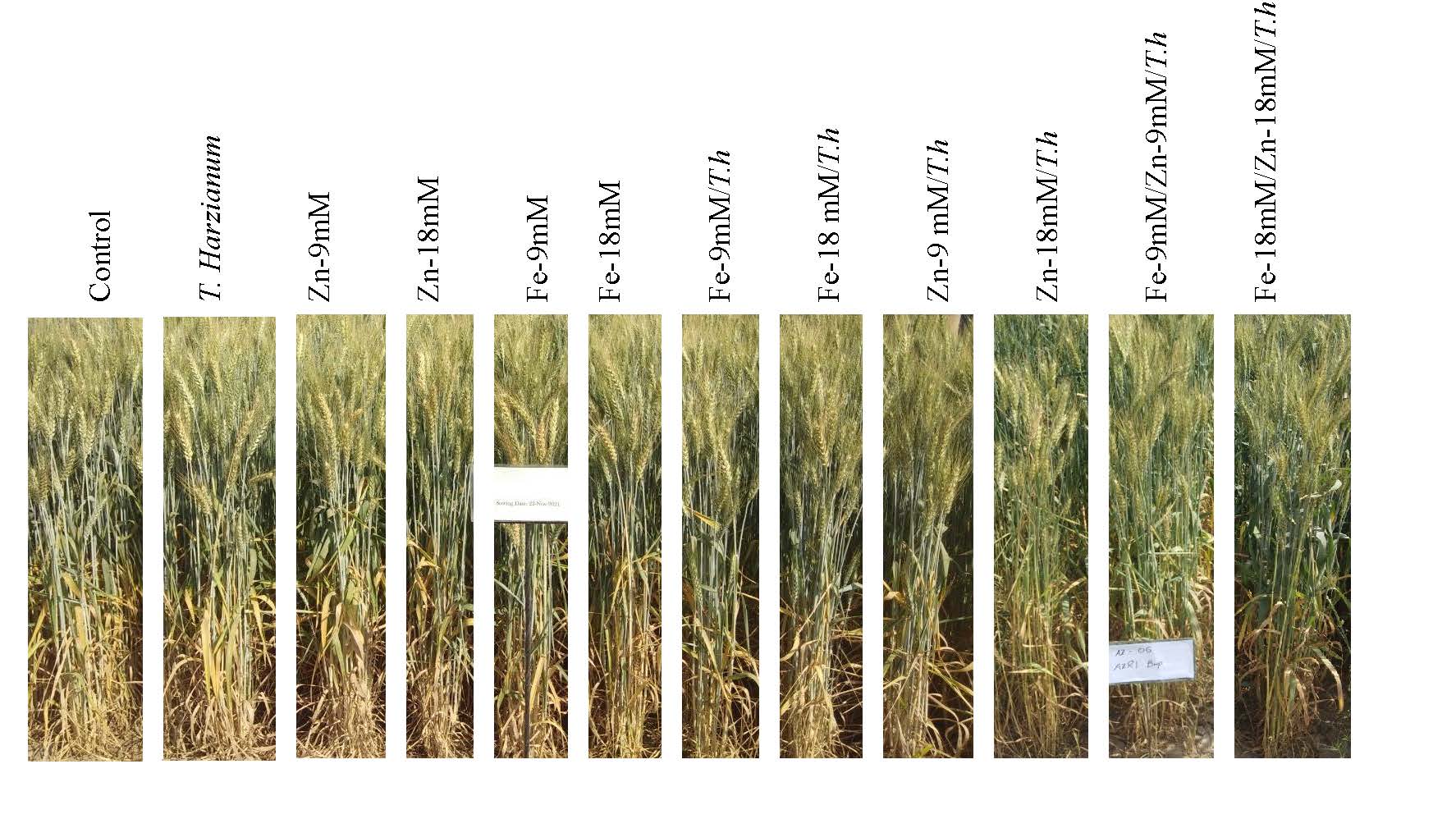


Figure S2: Morphological changes of wheat treated with Fe, Zn and *Trichoderma harzianum.*
